# Preoperative Systemic Immune–Inflammation Index (SII) as a Superior Predictor of Long-Term Survival Outcome in Patients With Stage I–II Gastric Cancer After Radical Surgery

**DOI:** 10.3389/fonc.2022.829689

**Published:** 2022-02-28

**Authors:** Kang He, Lixiang Si, Xiaohua Pan, Ling Sun, Yajing Wang, Jianwei Lu, Xiaohua Wang

**Affiliations:** The Department of Oncology, The Affiliated Cancer Hospital of Nanjing Medical University, and Jiangsu Cancer Hospital and Jiangsu Institute of Cancer Research, Nanjing, China

**Keywords:** stage I–II gastric cancer, hematological biomarkers, nutrition indices, inflammation indices, serum tumor markers, prognosis, dynamic nomogram

## Abstract

**Background:**

Systemic immune–inflammation index (SII), calculated by immunoinflammatory cell counts of peripheral blood, is considered a predictor of survival outcome in several solid tumors, including gastric cancer (GC). However, there is no study focusing on the prognostic value of SII in the early stage of GC. This study aims to compare prognostic prediction capabilities of several inflammatory indices, nutritional indices, and tumor markers to further verify the superior prognostic value of SII in stage I–II GC patients after surgery.

**Methods:**

In this study, 548 patients (358 in the training group and 190 in the validation group) with stage I–II GC after radical surgery were retrospectively analyzed. The peripheral blood indices of interest were SII, neutrophil-to-lymphocyte ratio (NLR), platelet-to-lymphocyte ratio (PLR), monocyte-to-lymphocyte ratio (MLR), advanced lung cancer inflammation index (ALI), systemic inflammation score (SIS), prognostic nutritional index (PNI), body mass index (BMI), albumin, carcinoembryonic antigen (CEA), cancer antigen 125 (CA125), carbohydrate-associated antigen 19-9 (CA19-9), and alpha-fetoprotein (AFP). The time-dependent receiver operating characteristic (t-ROC) curves and the area under the curve (AUC) were used to determine the optimal cutoff value and prognostic ability of each parameter. Kaplan–Meier curves and multivariable Cox regression models were used to evaluate independent prognostic factors. The nomogram was constructed based on the result of bidirectional stepwise regression model.

**Results:**

The optimal cutoff value of SII was 508.3. The 5-year overall survival rate of the low SII (SII-L) group was significantly higher than that of the high SII (SII-H) group (92% vs. 80%, P < 0.001), especially in the elderly and stage II patients (91% vs. 73%, P = 0.001; 86% vs. 67%, P = 0.003, respectively). The significant prognostic values of SII were consistent in most subgroups. In multivariate analysis, SII and CA19-9 were the only two independent prognostic hematology indices. The AUC value of SII (0.624) was greater than that of CA19-9 (0.528) and other prognostic parameters. Adding SII to the conventional model improved the predictive ability of 5-year overall survival as shown by the significantly increased net reclassification improvement (NRI) and integrated discrimination improvement (IDI) (P = 0.033, P = 0.053, respectively) and modestly improved consistency index (C-index) (increased by 1.6%). External validation of SII-based nomogram demonstrated favorable predictive performance and discrimination. In addition, interactive web dynamic nomogram was published to facilitate clinical use.

**Conclusion:**

SII is a simple but powerful index with a high predictive value to predict survival outcome in patients with stage I–II GC after radical operation. The SII-based nomogram can provide intuitive and accurate prognosis prediction of individual patients.

## Introduction

Gastric cancer (GC) is one of the most common malignant tumors and the fourth leading cause of cancer-related death worldwide according to the data from the World Health Organization (WHO) (https://gco.iarc.fr/). In China, approximately 478,508 new GC cases and 373,789 deaths occurred in 2020, ranking fifth and fourth all over the country, respectively. Although radical surgery is considered the best choice for patients with early and limited-stage cancer, about 35%–70% patients died within 5 years according to Surveillance, Epidemiology, and End Results (SEER) database (http://seer.cancer.gov/statfacts/html/stomach.html). Therefore, precise evaluation and prediction of individual prognosis of GC patients are the foundation for guiding treatment regimens and follow-up strategies.

The Tumor, Node, Metastasis (TNM) Staging System proposed by The American Joint Committee on Cancer (AJCC)/Union for International Cancer Control (UICC) is widely applied by clinical physicians to predict prognoses of GC patients. However, it is noticed that, in clinical practice, the heterogeneous survival prognosis is not uncommon even among patients with the same pathological stage, which cannot simply be explained by tumor TNM stage. Therefore, further studies are needed to discover better predictors of prognosis for patients with cancer.

Tumor-related inflammation plays an essential role in DNA damage, gene mutation, angiogenesis, proliferation, invasion, and metastasis of the tumor (1, 2). Tumor microenvironment is determined not only by the tumor itself but also by the host’s systemic immune-inflammatory response (3). Some inflammation-related indices, such as neutrophil-to-lymphocyte ratio (NLR), monocyte-to-lymphocyte ratio (MLR), platelet-to-lymphocyte ratio (PLR), advanced lung cancer inflammation index (ALI), and systemic immune–inflammation index (SII), which systemically calculate the status of immune-inflammatory cells, are considered to be promising prognostic indicators in several solid tumors, including GC (4–7). Due to the close relationship between immune function and cell metabolism and nutritional status (8), some studies have further found that some nutritional indices, such as albumin, body mass index (BMI), and prognostic nutritional index (PNI), are potential predictors of prognosis in patients with cancer (9, 10). In addition, serum tumor markers, recognized as prognostic factors, are widely used in assessing the effect of treatment, predicting prognosis and recurrence (11). SII, based on neutrophil (N), platelet (P), and lymphocyte (L) counts, has been regarded as a more promising prognostic index than other inflammation indices in recent years, but its prognostic value in early-stage GC remains unknown. Furthermore, there are still few studies comparing the prognostic value of inflammatory indices, nutritional indices, and serum tumor markers in patients with early-stage GC.

In the present study, we aim to compare multiple hematological indicators for predicting survival outcomes and further determine the superior prognostic value of SII in patients with stage I–II GC who are undergoing radical resection. In addition, SII-based nomogram is established to visualize risk factors and facilitate clinical decisions.

## Materials and Methods

### Ethics Statement

This research was reviewed and approved by the ethics committee of the Jiangsu Cancer Hospital, and all procedures were in compliance with the Helsinki declaration.

### Patients

**Figure 1:**
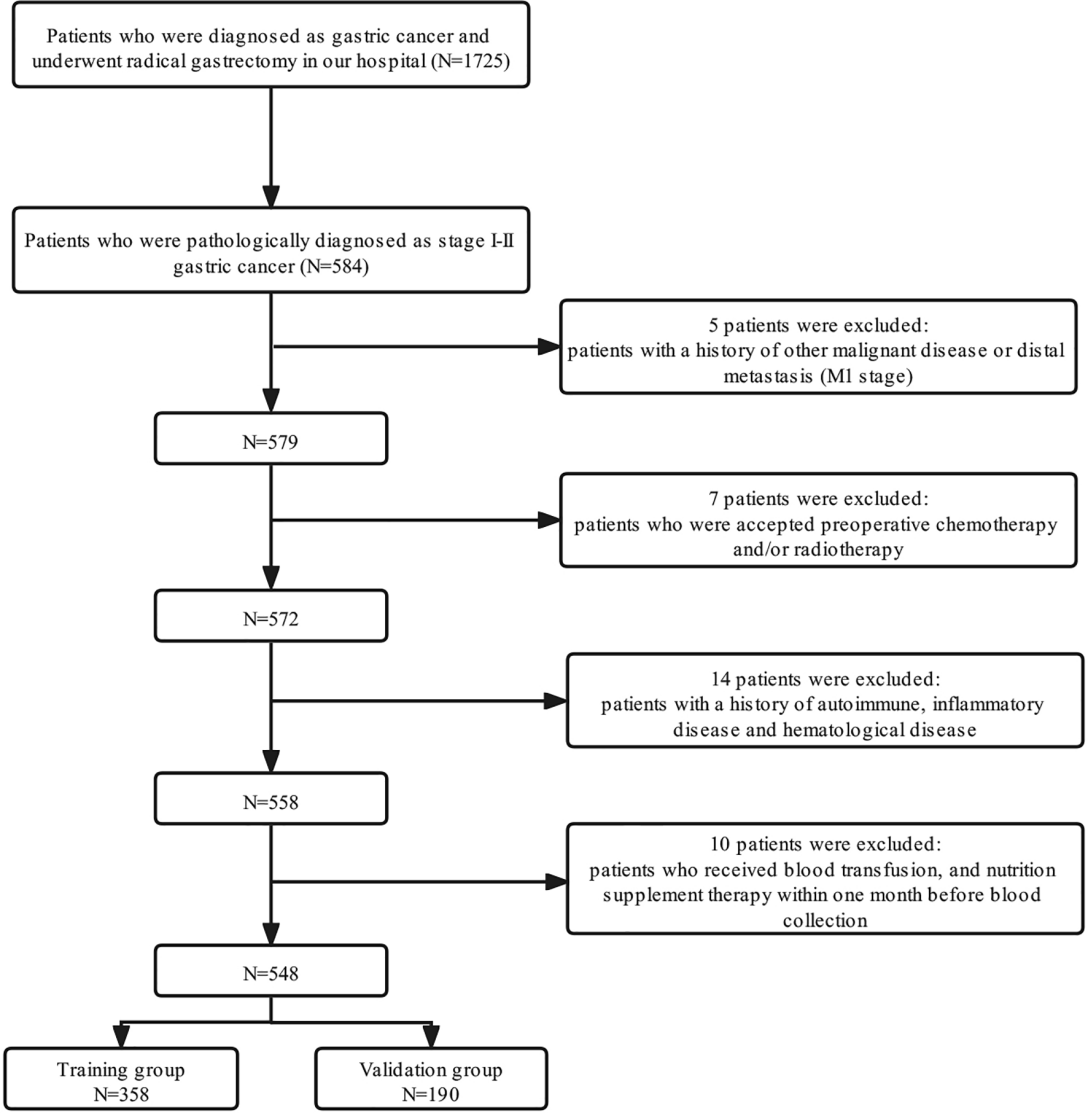
Flow diagram of the patient selection process.

A total of 1,725 patients who were diagnosed in our center as having GC from January 1, 2009, to March 31, 2016, were enrolled retrospectively. The inclusion criteria were as follows: 1) patients who underwent curative gastrectomy (R0) and had been pathologically diagnosed as stage I–II; 2) patients with Eastern Cooperative Oncology Group (ECOG) performance status between 0 and 2; 3) patients with complete clinicopathological and follow-up records. The exclusion criteria were as follows: 1) patients with distant metastases and/or other malignant diseases previously diagnosed; 2) patients who received neoadjuvant chemotherapy and/or radiotherapy; 3) patients with a history of autoimmune, inflammatory disease and hematological disease; 4) patients who received blood transfusion and nutrition supplement therapy within 1 month before blood collection. Finally, 548 patients who fulfilled the inclusion/exclusion criteria were included in our study; 358 patients diagnosed from January 1, 2009, to December 31, 2014, were assigned to the training group, and 190 patients diagnosed from January 1, 2015, to March 31, 2016, were assigned to the validation group. A detailed flowchart for the selection process is shown in **Figure 1**. After comprehensive consideration of therapy guidelines, pathological examination, radiological imaging tests, and patient willingness, the adjuvant chemotherapy regimens were decided, and the regimens were single-agent capecitabine.

### Data Collection

Clinicopathologic features included gender, age, history of smoking and alcohol intake, BMI, ECOG score, tumor site, pathological type, TNM stage. Nutrition-based indices, such as PNI and BMI, were calculated as follows: PNI = 10×albumin (g/dL) + 5×lymphocyte count (10^9^/L), BMI = body weight (kg)/height squared (m²). Inflammation-based indices, such as SII, NLR, PLR, MLR, and ALI, were calculated as follows: SII = platelet (P) × neutrophil (N)/lymphocyte (L); NLR = N/L; PLR = P/L; MLR = macrophagocyte (M)/L; ALI = BMI (kg/m²) × albumin (g/dl)/NLR. In addition, systemic inflammation score (SIS) was defined as follows: 0 point: both albumin ≥40 g/L and LMR ≥4.44; 1 point: patients with either albumin ≥40 g/L or LMR ≥4.44; and 2 points: both albumin <40 g/L and LMR <4.44. Serum tumor markers, such as carcinoembryonic antigen (CEA), carbohydrate-associated antigen 19-9 (CA19-9), cancer antigen 125 (CA125), and alpha-fetoprotein (AFP), were also analyzed in this study. According to the laboratory reference values, the cutoff values of albumin, CEA, AFP, CA125, and CA19-9 levels were 35 g/l, 3.5 ng/ml, 7 ng/ml, 35 U/ml, and 39 U/ml, respectively. Therefore, patients were further classified into the normal or the high group based on the cutoff value of each parameter. Blood samples for routine laboratory tests, such as complete blood count (CBC) and serum tumor markers, were collected within 7 days before surgery.

### Statistics

The time-dependent receiver operating characteristic (t-ROC) curves were used to determine the optimal cutoff value of inflammatory index and nutritional index. Patient clinicopathological characteristics were compared using t-test for continuous variables and chi-square test or Fisher’s exact test for categorical variables. Survival differences were compared by Kaplan–Meier method and log-rank test. Variables with P value <0.1 in the univariate survival analysis were included in multivariate Cox proportional hazards model to identify independent prognostic factors. In addition, BMI with marginal significance (P = 0.111) was also included in multivariate analysis because of its acknowledged prognostic value for GC and broad application in clinical practice. The area under the curve (AUC) was used to compare the prognostic ability of prognostic factors based on 5-year overall survival (OS). For model construction, the variables with P value <0.1 in the univariate survival analysis were candidates for the Cox proportional hazards bidirectional stepwise regression model to screen the risk factor used for the construction of the nomogram. Consistency index (C-index) and calibration curve were used to evaluate the discrimination ability of the nomogram. C-index, net reclassification improvement (NRI), and integrated discrimination improvement (IDI) were performed to assess whether there was a difference in diagnostic ability between conventional and SII-based model. Two-sided P values <0.05 were considered statistically significant. All statistical analyses were performed using SPSS 22.0 software (Chicago, IL, USA) and R software version 4.1.1 with the assistance of several R packages (including “survival,” “survminer,” “survivalROC,” “timeROC,” “forestmodel,” “rms,” “dplyr,” “DynNom,” “shiny,” “rsconnect,” “dcurves,” and “ggplot2”) (http://www.r-project.org/).

## Results

### Baseline Characteristics

A total of 358 cases in the training group (284 males and 74 females) with the median age of 61 years (range 56–67 years) were retrospectively analyzed in this study. All patients underwent radical gastrectomy and were pathologically diagnosed as having GC. Of all cases, 231 were stage I and 127 were stage II. Of the primary tumor location, 170 cases (47.5%) were located in the upper third of the stomach, 44 cases (12.3%) in the middle third, and 144 cases (40.2%) in the lower third. Here, 159 cases received postoperative adjuvant chemotherapy, and 199 cases did not. Detailed information is shown in **Table 1**.

**Table 1:** Baseline patient clinicopathological characteristics.

Characteristics	Cases (% of 358)
Sex	
Male	284 (79.3%)
Female	74 (20.7%)
Age (median, IQR)	61 (56–67)
Smoking history	
No	195 (54.5%)
Yes	163 (45.5%)
Drinking history	
No	208 (58.1%)
Yes	150 (41.9%)
BMI (kg/m²)	
<18.5	23 (6.4%)
18.5–25.0	220 (61.5%)
≥25.0	115 (32.1%)
ECOG score	
0	238 (66.5%)
1	88 (24.6%)
2	32 (8.9%)
Primary location	
Upper	170 (47.5%)
Middle	44 (12.3%)
Low	144 (40.2%)
Pathological type	
Adenocarcinoma	265 (74.0%)
Mucinous/rare carcinoma	93 (26.0%)
Histologic type	
Well and moderate	105 (29.3%)
Poor	253 (70.7%)
Neural invasion	
Negative	327 (91.3%)
Positive	31 (8.7%)
Lymphovascular invasion	
Negative	313 (87.4%)
Positive	45 (12.6%)
TNM stage	
I	231 (64.5%)
II	127 (35.5%)
Albumin	
Low	22 (6.1%)
Normal	336 (93.9%)
NLR	
Low	51 (14.2%)
High	307 (85.8%)
PLR	
Low	54 (15.1%)
High	304 (84.9%)
MLR	
Low	215 (60.1%)
High	143 (39.9%)
ALI	
Low	116 (32.4%)
High	242 (67.6%)
SII	
Low	267 (74.6%)
High	91 (25.4%)
PNI	
Low	197 (55.0%)
High	161 (45.0%)
SIS	
0	155 (43.3%)
1	146 (40.8%)
2	57 (15.9%)
CEA	
Normal	271 (75.7%)
High	87 (24.3%)
CA125	
Normal	351 (98.0%)
High	7 (2.0%)
CA19-9	
Normal	340 (95.0%)
High	18 (5.0%)
AFP	
Normal	338 (94.4%)
High	20 (5.6%)
Adjuvant chemotherapy	
Without	199 (55.6%)
With	159 (44.4%)

BMI, body mass index; ECOG, Eastern Cooperative Oncology Group; NLR, neutrophil to lymphocyte ratio; PLR, platelet to lymphocyte ratio; MLR, monocyte to lymphocyte ratio; ALI, advanced lung cancer inflammation index; SII, systemic immune-inflammation index; PNI, prognostic nutritional index; SIS, systemic inflammation score; CEA, carcinoembryonic antigen; CA125, cancer antigen 125; CA19-9, carbohydrate associated antigen 19-9; AFP, alpha-fetoprotein.

### The Optimal Cutoff Values for Inflammatory Index and Nutritional Index

The t-ROC curve was used to determine the optimal cutoff value for each inflammatory and nutritional index. The end point was 5-year OS rate. The optimal cutoff values for NLR, PLR, MLR, ALI, SII, and PNI were 1.2, 71.4, 0.24, 40.5, 508.3, 50.8, respectively. Patients were stratified to two groups (low and high group) based on the optimal cutoff value of each index.

### The Relationship Between Systemic Immune–Inflammation Index and Patient Characteristics

Finally, 267 patients in the low SII (SII-L) group and 91 patients in the high SII (SII-H) group were retrospectively analyzed. In terms of clinicopathological characteristics, better ECOG score and normal CA125 level tended to appear in patients in the SII-L group than patients in the SII-H group (P < 0.001, P = 0.013, respectively). In terms of other baseline characteristics, such as sex, age, BMI, tumor site, pathological type, TNM stage, albumin, and so on, no significant difference was observed between the SII-L and SII-H groups. In terms of serum tumor markers, the CEA, CA19-9, and AFP levels were similar between the two groups (P = 0.594, P = 0.785, P = 0.628, respectively). Details are shown in **Table 2**. In terms of inflammatory index, higher level of NLR, PLR, and MLR, lower level of ALI and PNI, and lower SIS score showed in SII-H group than those in SII-L group, and the differences were statistically significant (all P < 0.001) (**Table 3**).

**Table 2:** Baseline patient clinicopathological characteristics based on systemic immune–inflammation index (SII).

	SII-L	SII-H	P value
Sex			
Male	212 (79.4%)	72 (79.1%)	0.955
Female	55 (20.6%)	19 (20.9%)	
Age *	60.4 ± 8.8	62.2 ± 11.6	0.177
Smoking history			
No	142 (53.2%)	53 (58.2%)	0.403
Yes	125 (46.8%)	38 (41.8%)	
Drinking history			
No	149 (55.8%)	59 (64.8%)	0.132
Yes	118 (44.2%)	32 (35.2%)	
BMI (kg/m²)			
<18.5	18 (6.7%)	5 (5.5%)	0.321
18.5–25.0	169 (63.3%)	51 (56.0%)	
≥25.0	80 (30.0%)	35 (38.5%)	
ECOG score			
0	193 (72.3%)	45 (49.5%)	<0.001
1	53 (19.9%)	35 (38.5%)	
2	21 (7.9%)	11 (12.1%)	
Primary location			
Upper	118 (44.2%)	52 (57.1%)	0.102
Middle	35 (13.1%)	9 (9.9%)	
Low	114 (42.7%)	30 (33.0%)	
Pathological type			
Adenocarcinoma	199 (74.5%)	66 (72.5%)	0.707
Mucinous/rare carcinoma	68 (25.5%)	25 (27.5%)	
Histologic type			
Well and moderate	75 (28.1%)	30 (33.0%)	0.377
Poor	192 (71.9%)	61 (67.0%)	
Neural invasion			
Negative	243 (91.0%)	84 (92.3%)	0.704
Positive	24 (9.0%)	7 (7.7%)	
Lymphovascular invasion			
Negative	233 (87.3%)	80 (87.9%)	0.872
Positive	34 (12.7%)	11 (12.1%)	
TNM stage			
I	176 (65.9%)	55 (60.4%)	0.346
II	91 (34.1%)	36 (39.6%)	
Albumin			
Low	15 (5.6%)	7 (7.7%)	0.477
Normal	252 (94.4%)	84 (92.3%)	
CEA			
Normal	204 (76.4%)	67 (73.6%)	0.594
High	63 (23.6%)	24 (26.4%)	
CA125			
Normal	265 (99.3%)	86 (94.5%)	0.013^#^
High	2 (0.7%)	5 (5.5%)	
CA19-9			
Normal	254 (95.1%)	86 (94.5%)	0.785^#^
High	13 (4.9%)	5 (5.5%)	
AFP			
Normal	253 (94.8%)	85 (93.4%)	0.628
High	14 (5.2%)	6 (6.6%)	
Adjuvant chemotherapy			
Without	151 (56.6%)	48 (52.7%)	0.528
With	116 (43.4%)	43 (47.3%)	

*Two-tailed t tests of mean SD. ^#^Two-sided Fisher’s exact test, others are two-sided χ^2^ test.

BMI, body mass index; ECOG, Eastern Cooperative Oncology Group; CEA,carcinoembryonic antigen; CA125, cancer antigen 125; CA19-9, carbohydrate associated antigen 19-9; AFP, alpha-fetoprotein.

**Table 3:** Association between the systemic immune–inflammation index (SII) and hematological parameters.

	SII-L	SII-H	P value
NLR			
Low	51 (19.1%)	0 (0.0%)	<0.001
High	216 (80.9%)	91 (100.0%)	
PLR			
Low	54 (20.2%)	0 (0.0%)	<0.001
High	213 (79.8%)	91 (100.0%)	
MLR			
Low	186 (69.7%)	29 (31.6%)	<0.001
High	81 (30.3%)	62 (68.1%)	
ALI			
Low	48 (18.0%)	68 (74.7%)	<0.001
High	219 (82.0%)	23 (25.3%)	
PNI			
Low	130 (48.7%)	67 (73.6%)	<0.001
High	137 (51.3%)	24 (26.4%)	
SIS			
0	138 (51.7%)	17 (18.7%)	<0.001
1	103 (38.6%)	43 (47.3%)	
2	26 (9.7%)	31 (34.1%)	

NLR, neutrophil to lymphocyte ratio; PLR, platelet to lymphocyte ratio; MLR, monocyte to lymphocyte ratio; ALI, advanced lung cancer inflammation index; PNI, prognostic nutritional index; SIS, systemic inflammation score.

### Survival Analysis

**Figure 2:**
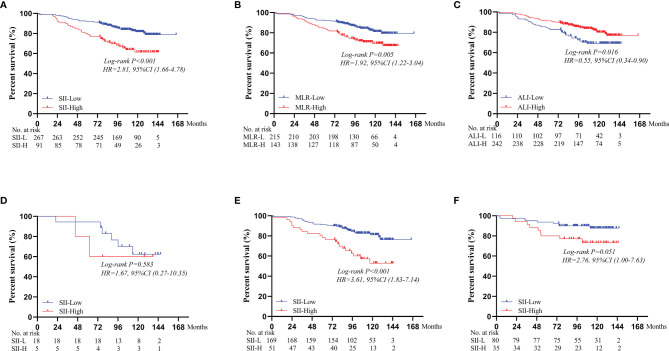
Subgroup survival analyses for different immunoinflammatory indices: **(A)** SII. **(B)** MLR. **(C)** ALI. Survival curves of OS comparing SII-L group and SII-H group according to the BMI score: **(D)** In the BMI < 18.5 group. **(E)** In the 18.5 ≤ BMI < 25.0 group. **(F)** In the BMI ≥ 25.0 group. SII, systemic immune-inflammation index; MLR, monocyte to lymphocyte ratio; ALI, advanced lung cancer inflammation index; BMI, body mass index.

As of December 31, 2020, no patients were lost to follow-up. The mean follow-up duration was 101 months (range, 2–166 months). In terms of survival outcomes, the 5-year OS rate of SII-L patients was statistically higher than that of SII-H patients (92% vs. 80%, P < 0.001; **Figure 2A**). Furthermore, we also found that the 5-year OS rate of low MLR (MLR-L) patients was statistically higher than that of high MLR (MLR-H) patients (92% vs. 85%, P = 0.005; **Figure 2B**), and high ALI (ALI-H) patients had a significantly higher 5-year OS rate than that of low ALI (ALI-L) patients (91% vs. 85%, P = 0.016; **Figure 2C**).

Univariate analysis showed that age (P < 0.001), tumor site (P = 0.005), TNM stage (P < 0.001), MLR (P = 0.006), ALI (P = 0.018), SII (P < 0.001), CA19-9 (P = 0.019), and AFP (P = 0.014) were statistical prognostic factors. Multivariate Cox survival analysis found that SII (P = 0.009), CA19-9 (P = 0.039), age (P < 0.001), and TNM stage (P < 0.001) were independent prognostic factors. Details are shown in **Table 4**.

**Table 4:** Univariate and multivariate analyses of overall survival according to clinicopathologic factors.

	Univariate analysis		Multivariate analysis	
	HR (95% CI)	P value	HR (95% CI)	P value
Sex				
Male vs. Female	0.989 (0.570–1.716)	0.970		
Age, years	1.064 (1.037–1.093)	<0.001	1.057 (1.025–1.089)	<0.001
Smoking history				
No vs. Yes	1.124 (0.719–1.757)	0.609		
Drinking history				
No vs. Yes	0.802 (0.506–1.272)	0.348		
BMI (kg/m²)				
<18.5	1.000	0.111	1.000	0.171
18.5–25.0	0.680 (0.323–1.434)		0.604 (0.273–1.335)	
≥25.0	0.437 (0.190–1.005)		0.420 (0.167–1.055)	
ECOG score				
0	1.000	0.516		
1	1.340 (0.814–2.207)			
2	1.107 (0.500–2.450)			
				
Primary location				
Upper	1.000	0.005	1.000	0.114
Middle	0.786 (0.397–1.553)		0.998 (0.483–2.062)	
Low	0.415 (0.244–0.706)		0.560 (0.318–0.988)	
Pathological type				
Adenocarcinoma vs. Mucinous/rare carcinoma	0.906 (0.540–1.522)	0.710		
Histologic type				
Well/moderate vs. Poor	1.003 (0.615–1.634)	0.991		
Neural invasion				
Negative vs. Positive	1.729 (0.889–3.363)	0.106		
Lymphovascular invasion				
Negative vs. Positive	1.483 (0.817–2.693)	0.195		
TNM stage				
I vs. II	2.556 (1.630–4.009)	<0.001	2.728 (1.681–4.426)	<0.001
Albumin				
Low vs. Normal	1.024 (0.414–2.535)	0.959		
NLR				
Low vs. High	1.422 (0.683–2.957)	0.347		
PLR				
Low vs. High	2.140 (0.930–4.924)	0.074	1.360 (0.563–3.285)	0.494
MLR				
Low vs. High	1.876 (1.198–2.938)	0.006	1.673 (0.738–3.794)	0.218
ALI				
Low vs. High	0.580 (0.370–0.909)	0.018	1.338 (0.735–2.436)	0.341
SII				
Low vs. High	2.359 (1.499–3.713)	<0.001	2.270 (1.230–4.188)	0.009
PNI				
Low vs. High	0.938 (0.596–1.477)	0.783		
SIS				
0	1.000	0.075	1.000	0.468
1	1.210 (0.722–2.028)		0.627 (0.280–1.402)	
2	1.960 (1.090–3.527)		0.541 (0.196–1.498)	
CEA				
Normal vs. High	1.591 (0.987–2.563)	0.057	1.174 (0.706–1.951)	0.536
CA125				
Normal vs. High	2.147 (0.676–6.812)	0.195		
CA19-9				
Normal vs. High	2.396 (1.152–4.982)	0.019	2.270 (1.043–4.942)	0.039
AFP				
Normal vs. High	2.382 (1.188–4.778)	0.014	1.701 (0.807–3.586)	0.163
Adjuvant chemotherapy				
Without vs. With	0.728 (0.457–1.159)	0.181		

CI, confidence interval; HR, hazard ratio; BMI, body mass index; ECOG, Eastern Cooperative Oncology Group; NLR, neutrophil to lymphocyte ratio; PLR, platelet to lymphocyte ratio; MLR, monocyte to lymphocyte ratio; ALI, advanced lung cancer inflammation index; SII, systemic immune-inflammation index; PNI, prognostic nutritional index; SIS, systemic inflammation score; CEA, carcinoembryonic antigen; CA125, cancer antigen 125; CA19-9, carbohydrate associated antigen 19-9; AFP, alphafetoprotein.

### Subgroup Analysis of the Prognostic Value of Systemic Immune–Inflammation Index

**Figure 3:**
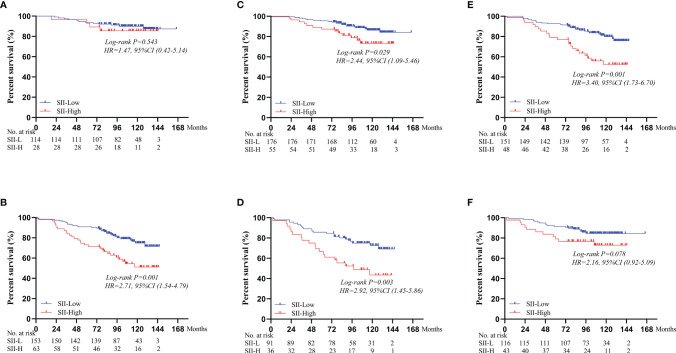
Survival curves of OS comparing SII-L group and SII-H group based on the age, TNM stage, and adjuvant chemotherapy status. **(A)** In the non-elderly group. **(B)** In the elderly group. **(C)** In the stage I group. **(D)** In the stage II group. **(E)** In the non-adjuvant group. **(F)** In the adjuvant group. SII, systemic immune-inflammation index.

**Figure 4:**
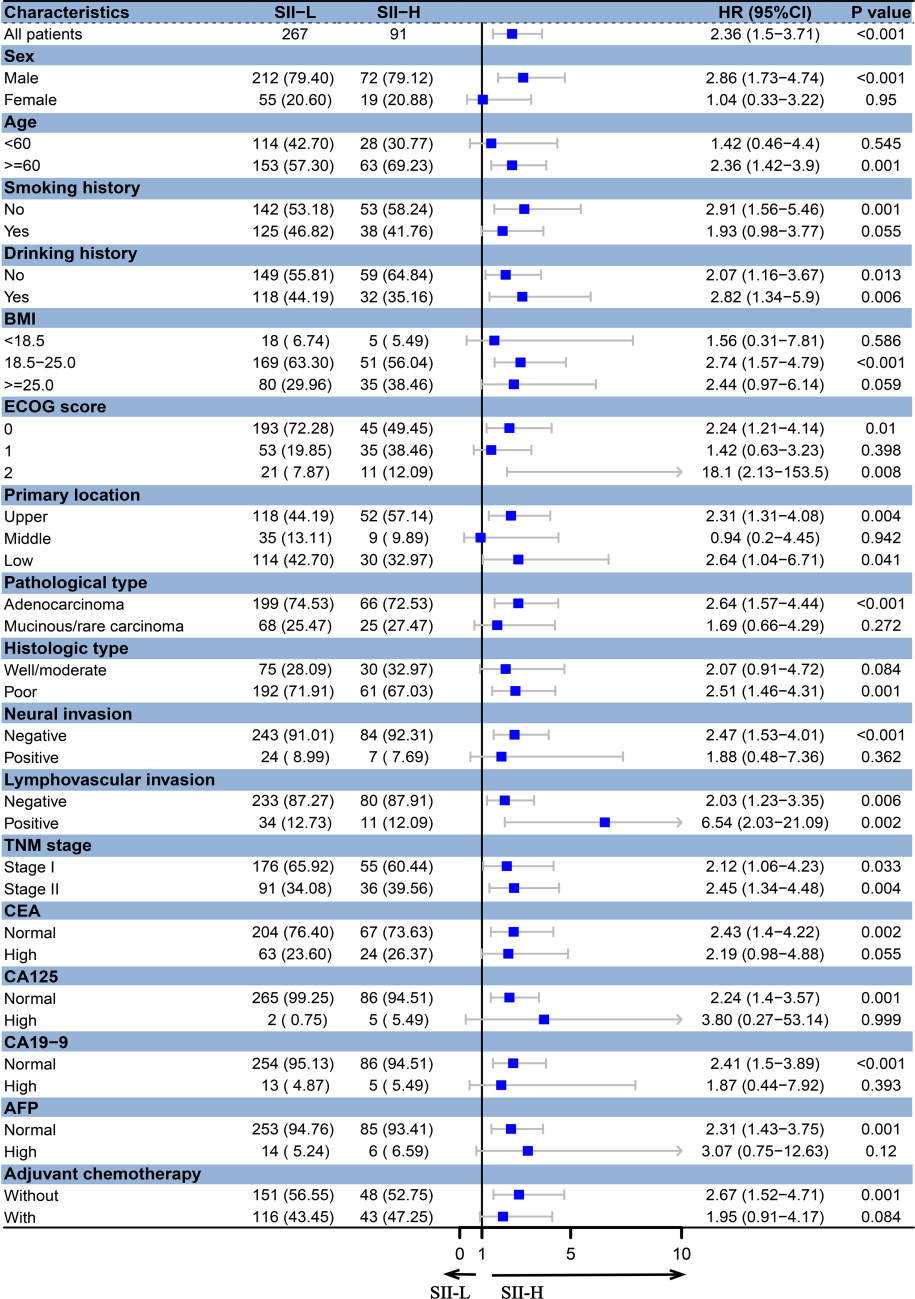
Forest plots for subgroup analyses of SII in stage I–II GC. BMI, body mass index; ECOG, Eastern Cooperative Oncology Group; CEA, carcinoembryonic antigen; CA125, cancer antigen 125; CA19-9, carbohydrate associated antigen 19-9; AFP, alpha-fetoprotein.

Based on different BMI scores, patients were divided into three groups (BMI < 18.5, 18.5 ≤ BMI < 25.0, and BMI ≥ 25.0). In the BMI < 18.5 group, there was no significant difference in the 5-year OS rate between SII-L and SII-H group (94% vs. 80%, P = 0.583; **Figure 2D**). In the 18.5 ≤ BMI < 25.0 group, the 5-year OS rate in SII-L was significantly improved than that in SII-H (91% vs. 80%, P < 0.001; **Figure 2E**). In the BMI ≥ 25.0 group, there was no significant difference in the 5-year OS rate between SII-L and SII-H groups (94% vs. 80%, P = 0.051; **Figure 2F**). Based on age, patients were divided into the non-elderly group (aged < 60 years) and the elderly group (aged ≥ 60 years). In the non-elderly group, there was no obvious difference in the 5-year OS rate between the SII-L group and the SII-H group (94% vs. 96%, P = 0.543; **Figure 3A**). In the elderly group, postoperative survival was significantly longer in the SII-L group than that in the SII-H group (91% vs. 73%, P = 0.001; **Figure 3B**). Based on the TNM stage, patients were divided into stage I and stage II group. The 5-year OS rate in SII-L was statistically higher than that in SII-H in both stage I group (96% vs. 89%, P = 0.029; **Figure 3C**) and stage II group (86% vs. 67%, P = 0.003; **Figure 3D**). Based on adjuvant chemotherapy status, patients were further divided into adjuvant and non-adjuvant group. In the non-adjuvant group, the survival outcome of SII-H patients was significantly worse than that of SII-L patients (79% vs. 93%, P = 0.001; **Figure 3E**). However, in the adjuvant group, no noticeable survival difference was observed between the two groups (81% vs. 91%, P = 0.078; **Figure 3F**). After comparing the prognostic value of SII in the subgroup of each clinicopathological factor, we found that the prognostic value of SII was consistent in most subgroups (**Figure 4**).

### Predictive Ability of Systemic Immune–Inflammation Index

**Figure 5:**
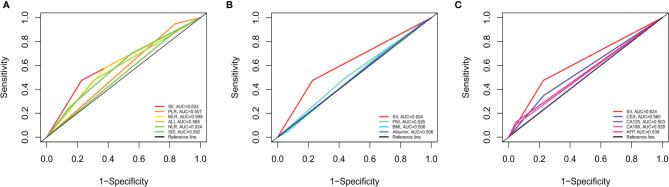
Predictive abilities of SII and other hematological indices for OS examined using t-ROC curves. **(A)** Predictive ability of SII and immunoinflammatory indices. **(B)** Predictive ability of SII and nutritional indices. **(C)** Predictive ability of SII and tumor markers. NLR, neutrophil to lymphocyte ratio; PLR, platelet to lymphocyte ratio; MLR, monocyte to lymphocyte ratio; ALI, advanced lung cancer inflammation index; SII, systemic immune-inflammation index; SIS, systemic inflammation score; PNI, prognostic nutritional index; BMI, body mass index; CEA, carcinoembryonic antigen; CA125, cancer antigen 125; CA19-9, carbohydrate associated antigen 19-9; AFP, alpha-fetoprotein.

**Figure 6:**
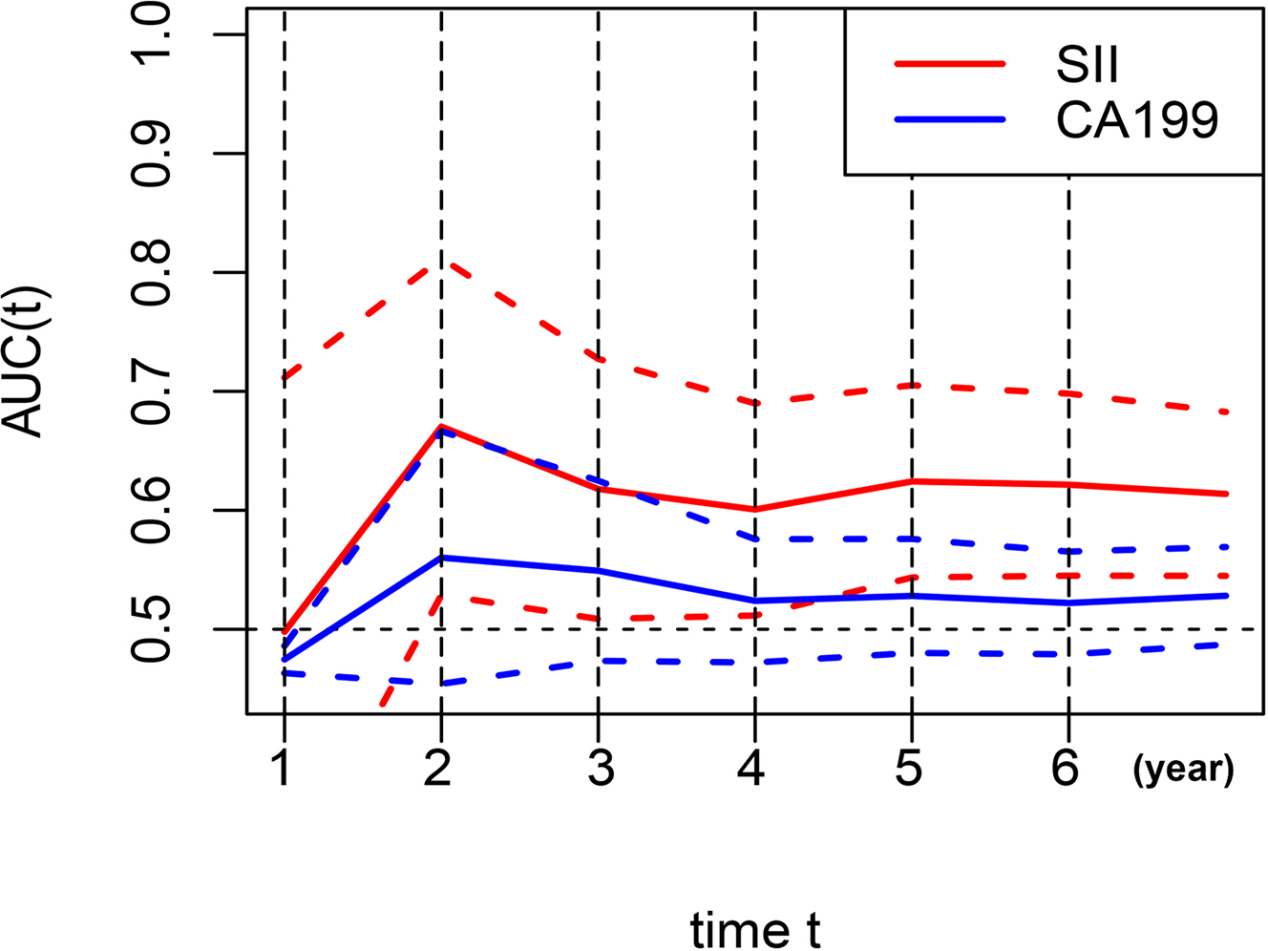
Dynamic change for predictive abilities of SII and CA19-9 during the observation period. SII, systemic immune-inflammation index; CA19-9, carbohydrate associated antigen 19-9.

AUC was performed to evaluate the predictive values of prognostic factors. The results indicated that the predictive ability of SII (0.624, 95% CI 0.544–0.705) was better than other components, as follows: inflammatory indices (**Figure 5A**): NLR (0.524, 95% CI 0.442–0.607, P = 0.015), PLR (0.557, 95% CI 0.517–0.596, P = 0.106), MLR (0.599, 95% CI 0.518–0.680, P = 0.599), ALI (0.585, 95% CI 0.504–0.667, P = 0.235), and SIS (0.592, 95% CI 0.505–0.679, P = 0.487); nutritional indices (**Figure 5B**): PNI (0.528, 95% CI 0.446–0.611, P = 0.171), BMI (0.506, 95% CI 0.427–0.584, P = 0.051), and albumin (0.506, 95% CI 0.470–0.543, P = 0.004); tumor markers (**Figure 5C**): CEA (0.560, 95% CI 0.483–0.638, P = 0.253), CA125 (0.503, 95% CI 0.478–0.528, P = 0.003), CA19-9 (0.528, 95% CI 0.480–0.576, P = 0.043), and AFP (0.539, 95% CI 0.486–0.592, P = 0.090). Furthermore, in several blood parameters analyzed above, SII and CA19-9 were the only two independent prognostic factors in the multivariate Cox analysis. Time-dependent AUC curves of SII and CA19-9 were generated to further compare the predictive accuracy of OS throughout the observation period. The result showed that SII was superior to CA19-9 for predicting OS during the entire observation period (**Figure 6**). 

### Comparison Between the Conventional Nomogram and Systemic Immune–Inflammation Index-Based Nomogram

**Figure 7:**
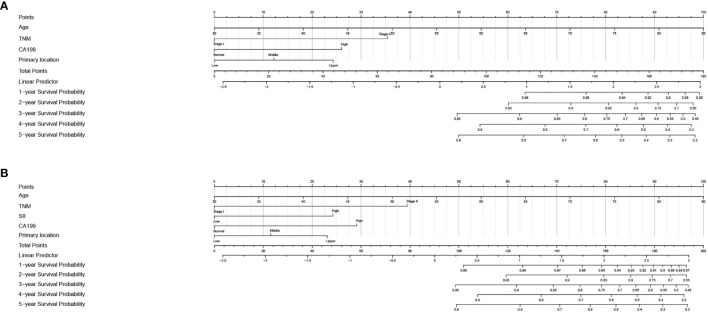
Nomogram for 1–5-year overall survival in stage I–II GC. **(A)** Conventional nomogram with significant clinical factors. **(B)** SII-based survival nomogram with SII and significant clinical factors. SII, systemic immune-inflammation index; CA19-9, carbohydrate associated antigen 19-9.

**Figure 8:**
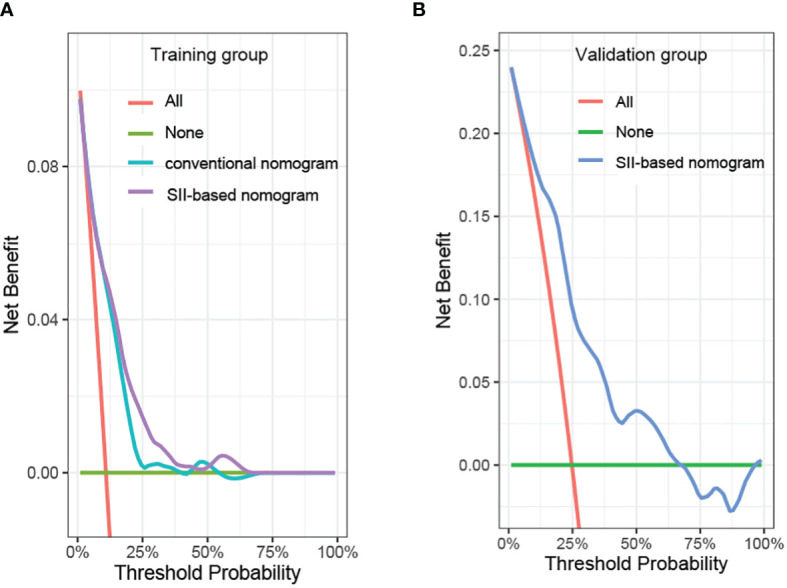
Decision curve analysis of the prediction model in the **(A)** training group and **(B)** validation group. SII, systemic immune-inflammation index.

**Figure 9:**
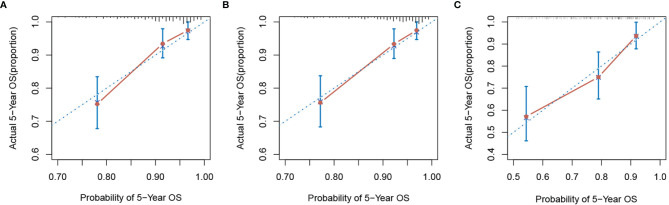
The calibration curve of nomograms for predicting 5-year overall survival. **(A)** Conventional nomogram and **(B)** SII-based nomogram in the training group. **(C)** SII-based nomogram in the validation group. SII, systemic immune-inflammation index.

In order to further predict 1–5-year OS of stage I–II GC patients after surgery, nomograms were established based on the results of the Cox proportional hazards bidirectional stepwise regression model (including age, TNM stage, primary location, SII, and CA19-9) (**Figures 7A, B**). The C-index of the conventional nomogram (including age, TNM stage, primary location, and CA19-9) was 0.733. The C-index of SII-based nomogram (including age, TNM stage, primary location, SII, and CA19-9) was 0.745. Adding SII to the conventional model improved the predictive ability of 5-year OS as shown by the statistically improved net reclassification improvement (NRI) of 0.249 (P = 0.033) and integrated discrimination improvement (IDI) of 0.027 (P = 0.053) and modestly improved C-index of 0.745 (P = 0.261); detailed information is shown in **Table 5**. Decision curve analyses also confirmed that the clinical net benefit for SII-based nomogram at the time point of 5-year OS was better than that of conventional one within the threshold probabilities of 13%–63% (**Figure 8A**). In addition, compared with the calibration curve of conventional nomogram, the calibration curve of SII-based nomogram showed better consistency between predictions and actual observations for the probability of 5-year OS (**Figures 9A, B**).

**Table 5:** Evaluation of predictive models for overall survival.

	C-index (95% CI)	P value	NRI (95% CI)	P value	IDI (95% CI)	P value
Conventional model	0.733 (0.705–0.761)	0.261	Ref	0.033	Ref	0.053
Conventional model + SII	0.745 (0.717–0.773)		0.249 (0.022–0.375)		0.027 (0.000–0.057)	

Finally, in addition to the traditional nomogram, we also published a dynamic nomogram (based on SII) that can predict the prognosis of patients through a simple operation on the website (https://hekang.shinyapps.io/DynNomapp/).

### External Validation of Systemic Immune–Inflammation Index-Based Model

Patient baseline characteristics in the training dataset were basically consistent with those of the validation dataset (**Table 6**). As of December 31, 2021, no patients of the validation group were lost to follow-up. The mean follow-up duration was 65 months (range, 4–85 months). The performance of the SII-based model was validated using the external dataset; the C-index of SII-based model was 0.737. Decision curve analysis of SII-based nomogram at the time point of 5-year OS is shown in **Figure 8B**. Most importantly, the calibration curve (**Figure 9C**) showed that the predicted 5-year OS of the validation dataset closely corresponded to the actual survival outcome.

**Table 6:** Baseline patient clinicopathological characteristics based on different datasets.

	Training dataset	Validation dataset	P value
Sex			
Male	284 (79.3%)	142 (74.7%)	0.219
Female	74 (20.7%)	48 (25.3%)	
Age *	60.9 ± 9.6	61.6 ± 10.0	0.408
Smoking history			
No	195 (54.5%)	109 (57.4%)	0.516
Yes	163 (45.5%)	81 (42.6%)	
Drinking history			
No	208 (58.1%)	107 (56.3%)	0.688
Yes	150 (41.9%)	83 (43.7%)	
BMI (kg/m²)			
<18.5	23 (6.4%)	9 (4.7%)	0.679
18.5–25.0	220 (61.5%)	116 (61.1%)	
≥25.0	115 (32.1%)	65 (34.2%)	
ECOG score			
0	238 (66.5%)	125 (65.8%)	0.976
1	88 (24.6%)	47 (24.7%)	
2	32 (8.9%)	18 (9.5%)	
Primary location			
Upper	170 (47.5%)	69 (36.3%)	<0.001
Middle	44 (12.3%)	50 (26.3%)	
Low	144 (40.2%)	71 (37.4%)	
Pathological type			
Adenocarcinoma	265 (74.0%)	125 (65.8%)	0.043
Mucinous/rare carcinoma	93 (26.0%)	65 (34.2%)	
Histologic type			
Well and moderate	105 (29.3%)	42 (22.1%)	0.069
Poor	253 (70.7%)	148 (77.9%)	
Neural invasion			
Negative	327 (91.3%)	167 (87.9%)	0.198
Positive	31 (8.7%)	23 (12.1%)	
Lymphovascular invasion			
Negative	313(87.4%)	163 (85.8%)	0.588
Positive	45(12.6%)	27 (14.2%)	
TNM stage			
I	231 (64.5%)	76 (40.0%)	<0.001
II	127 (35.5%)	114 (60.0%)	
Albumin			
Low	22 (6.1%)	8 (4.2%)	0.343
Normal	336 (93.9%)	182 (95.8%)	
CEA			
Normal	271 (75.7%)	143 (75.3%)	0.910
High	87 (24.3%)	47 (24.7%)	
CA125			
Normal	351 (98.0%)	181 (95.3%)	0.066
High	7 (2.0%)	9 (4.7%)	
CA19-9			
Normal	340 (95.0%)	174 (91.6%)	0.117
High	18 (5.0%)	16 (8.4%)	
AFP			
Normal	338 (94.4%)	179 (94.2%)	0.922
High	20 (5.6%)	11 (5.8%)	
Adjuvant chemotherapy			
Without	199 (55.6%)	102 (53.7%)	0.670
With	159 (44.4%)	88 (46.3%)	
SII			
Low	267 (74.6%)	114 (60.0%)	<0.001
High	91 (25.4%)	76 (40.0%)	
NLR			
Low	51 (14.2%)	25 (13.2%)	0.726
High	307 (85.8%)	165 (86.8%)	
PLR			
Low	54 (15.1%)	17 (8.9%)	0.042
High	304 (84.9%)	173 (91.1%)	
MLR			
Low	215 (60.1%)	114 (60.0%)	0.990
High	143 (39.9%)	76 (40.0%)	
ALI			
Low	116 (32.4%)	68 (35.8%)	0.424
High	242 (67.6%)	122 (64.2%)	
PNI			
Low	197 (55.0%)	93 (48.9%)	0.175
High	161 (45.0%)	97 (51.1%)	
SIS			
0	155 (43.3%)	82 (43.2%)	0.998
1	146 (40.8%)	78 (41.1%)	
2	57 (15.9%)	30 (15.8%)	

*Two-tailed t tests of mean SD, others are two-sided χ^2^ test.

BMI, body mass index; ECOG, Eastern Cooperative Oncology Group; NLR, neutrophil to lymphocyte ratio; PLR, platelet to lymphocyte ratio; MLR, monocyte to lymphocyte ratio; ALI, advanced lung cancer inflammation index; SII, systemic immune-inflammation index; PNI, prognostic nutritional index; SIS, systemic inflammation score; CEA, carcinoembryonic antigen; CA125, cancer antigen 125; CA19-9, carbohydrate associated antigen 19-9; AFP, alpha-fetoprotein.

## Discussion

Though the incidence of GC was gradually decreasing in China, it was still one of the most common cancer types of the digestive tract. Early diagnosis and early treatment were key principles during the whole period of cancer treatment and follow-up. For GC patients who received curative gastrectomy, pathological TNM (pTNM) stage was regularly used as a critical standard to predict prognosis and guide therapy regimens. However, in clinical practice, the survival outcomes for GC patients were diverse even in the same disease stage. One possible reason might be that the TNM stage can only reflect the biological characteristics of the primary tumor but not tumor and host inflammatory response (12). It was generally believed that a great number of inflammatory mediators could induce an inflammatory cascade and tissue atrophy and promote tumor proliferation and metastasis (13, 14). Therefore, the systemic inflammatory response was closely related to tumorigenesis and the prognosis of cancer patients. By comparing indices such as nutrition indices, inflammation indices, and serum tumor markers, this study aimed to discover the best prognostic factors in patients with early-stage GC. We also established a nomogram to intuitively assess an individualized survival outcome and guide clinical practice.

Tumor cells played an important role in the formation of proinflammatory mediators. Systemic inflammation promoted tumor invasion and progression by reducing apoptosis and promoting angiogenesis (15, 16). Peripheral blood cells, like neutrophils, platelets, and lymphocytes, were regarded as systemic immune and inflammatory cells in the body. Neutrophils not only inhibited the lymphocyte-mediated immune system (mainly T-cell activation) to promote tumor proliferation by secreting numerous inflammatory mediators, such as interleukin (IL)-6, IL-8, and vascular epithelial growth factor (17, 18) but also enhanced adhesion and distant metastasis of circulating tumor cells (CTCs) (19, 20). Lymphocytes played a core role in cellular immune surveillance and suppression of cancer cell proliferation and migration by inducing the growth of cytotoxic cells and secreting cytokines (15). In addition, previous experimental evidence showed that platelets could protect CTCs from shear stress in the circulation and enhanced epithelial–mesenchymal transition (21, 22). Platelets also allowed tumor cells to escape from the immune surveillance to distant organs by releasing ATP and relaxing the endothelial barrier (23). In recent years, several studies found that some inflammation-based indices, such as NLR, PLR, MLR, ALI, SII, and SIS, were calculated based on the combination of the blood components mentioned above and served as prognostic factors in many different types of cancer (4–7), where NLR and PLR were the most well-studied prognostic indices in GC. In a meta-analysis published in 2020, Kim et al. (24) recruited 18,348 patients and found that NLR was an independent factor for GC patients, regardless of race, tumor stage, and chemotherapy strategy. Cao et al. (25) confirmed in a meta-analysis that elevated PLR was related to poor OS in GC patients. Based on an integrated index of NLR and PLR, SII was recently recognized as a better predictor of the clinical prognosis in hepatocellular carcinoma (26), non-small cell lung cancer (27), colorectal cancer (28), and GC (29, 30). Due to high metabolism and proliferation of tumor cells, patients with cancer were more prone to malnutrition that correlated with damaged immune function and increased mortality (31). Several studies showed that some nutritional indices, such as albumin, BMI, and PNI, were also related to prognosis in GC patients after gastrectomy (9, 32). As far as we know, this was the first study to compare multiple hematological markers, such as immunoinflammatory indices, nutritional indices, and serum tumor markers, to find the optimal prognostic factors for stage I–II GC patients after radical gastrectomy.

In our study, the optimal cutoff value of several blood indices was analyzed through t-ROC curve according to 5-year OS, where the optimal cutoff value for SII, NLR, PLR, MLR, ALI, and PNI were 508.3, 1.2, 71.4, 0.24, 40.5, and 50.8, respectively. Correlation analyses showed that the ECOG score of patients in the SII-H group was poorer than that in the SII-L group, while the level of CA125 of patients in the SII-H group was higher than that in the SII-L group, partly validating the hypothesis that the increased inflammatory response might promote tumor metabolism and proliferation to cause the hypermetabolic state of patients.

Multivariate survival analyses showed that SII and CA19-9 were the only two independent prognostic factors for OS in several hematological indices included in this study. For further evaluating and comparing the predictive abilities of several indices based on the AUC values, these indices were divided into three groups (inflammatory parameters group, nutritional parameters group, and tumor markers group) and compared with SII, respectively. We also compared the predictive abilities of SII and CA19-9 in each year of the observation period and finally demonstrated that SII was the most valuable predictor for long-term survival outcome. Consistent with the results of previous studies, Shi et al. (30) confirmed that SII was the most effective predictor of OS compared to NLR, PLR, and MLR in GC patients; Zhu et al. (33) also reported on the superior predictive abilities of SII in patients with signet-ring cell GC.

In subgroup analyses, we found that, regardless of TNM stage, the survival outcomes of SII-H patients were significantly poorer than those of SII-L patients, and the survival differences were more significant in stage II group. The results were consistent with the findings of a previous study (29). When patients were stratified based on BMI scores, we found that in the normal-weight (BMI = 18.5–25.0) group, the survival time of GC patients in the SII-L group was statistically longer than that of patients in the SII-H group, while no statistical survival difference was found in wasting (BMI <18.5) group and overweight (BMI ≥25.0) group. This observation suggested that clinicians should realize the interaction between inflammation and nutrition status in cancer patients and pay more attention to inflammatory status of GC patients with normal weight and improve the nutritional status of non-normal-weight patients. In addition, SII was a prognostic factor in the elderly patient group, but not in the non-elderly patient group, which was consistent with previous studies (34). The elderly tended to develop cancer-related inflammation and immune defects, and regardless of the presence of cancer, the possibility of immunodeficiency increased with age (35, 36). SII might be a potential prognostic factor in aged GC patients, especially in today’s growing aging society.

In clinical practice, each variable alone could only play a limited prognostic ability to assess the risk of death because of the complex and heterogeneous nature of cancer. The nomogram seemed to be a good way to improve the prediction ability and facilitate clinical application that could integrate several risk factors into the prediction and considered the weight of each variable. With the nomogram, we could predict the 1–5-year OS of each patient by adding up the total scores shown in the bottom scale. In our study, variables with P values <0.1 in the univariate analysis were candidates for the Cox proportional hazards bidirectional stepwise regression model; we finally screened five variables (including age, TNM stage, primary location, SII, and CA19-9) to construct the nomogram. In order to validate the contribution ability of SII to model performance, we developed two nomograms, one was conventional nomogram (including age, TNM stage, primary location, and CA19-9) and the other was SII-based nomogram (including age, TNM stage, primary location, CA19-9, and SII). Compared to the conventional nomogram, improved predictive ability of SII-based nomogram was shown by the significantly increased NRI and IDI and modestly improved C-index. Decision curve analyses were also performed in our study to compare the net benefit between two nomograms; the results showed that the net benefit of the SII-based nomogram was better than that of the conventional nomogram at the time point of 5-year OS. The calibration curve also showed good predictive performance of the SII-based nomogram.

To further assess both performance and generalizability of the SII-based nomogram, we verified the model in an external validation queue. According to the results of C-index and calibration curve, the SII-based nomogram showed a stable and good performance in the external dataset. Furthermore, on the basis of the traditional nomogram, we also established a dynamic nomogram that can predict the survival outcome of individual patients. By dragging the slider to change the corresponding parameters, survival curve, predicted values, and corresponding 95% confidence intervals of individual patients were displayed automatically, which were convenient for broad clinical application. Prognosis of GC patients became worse as cumulative scores increased; patients with higher scores might have higher inflammatory status and poorer survival outcomes. This nomogram, to a certain extent, could be used as a reference for predicting the prognosis of GC patients and guiding individualized therapy strategy.

There were some limitations in our study. Firstly, although the sample size of the current study was already the largest in existing studies focusing on the prognostic value of SII in patients with early-stage GC, due to the nature of single-center retrospective study, the results might be affected by selection bias. Secondly, there was no consensual cutoff value for most inflammation and nutrition indices. In our retrospective studies, optimal cutoff values of these indices, such as SII, NLR, PLR, ALI, and PNI, were determined through t-ROC curve. Therefore, further large-sample prospective studies are needed to determine the universal cutoff value and validate the results of this study. Finally, blood samples were only collected at a single time point, so further study should collect blood samples in multiple time points and evaluate dynamic changes of SII.

## Conclusion

By comparing several inflammatory indices, nutritional indices, and serum tumor markers, this study confirms that SII has a better predictive value of OS in patients with stage I–II GC after radical surgery, especially in the elderly and stage II patients. In addition, the prognostic value of SII is also consistent in most subgroups. The SII-based nomogram can provide intuitive and accurate prognosis prediction of individual patients. In conclusion, as a low-cost, noninvasive, easy-to-assess, and reproducible prognostic parameter, SII can be a simple but powerful index for identifying the different prognoses of stage I–II GC patients.

## Data Availability Statement

The raw data supporting the conclusions of this article will be made available by the authors without undue reservation.

## Ethics Statement

The studies involving human participants were reviewed and approved by the ethics committee of the Jiangsu Cancer Hospital. Written informed consent for participation was not required for this study in accordance with national legislation and institutional requirements.

## Author Contributions

KH collected the data and wrote the paper. XW and JL reviewed the paper. LS and XP developed the methodology. LXS and YW analyzed the study data through statistics software. All authors contributed to the article and approved the submitted version.

## Conflict of Interest

The authors declare that the research was conducted in the absence of any commercial or financial relationships that could be construed as a potential conflict of interest.

## Publisher’s Note

All claims expressed in this article are solely those of the authors and do not necessarily represent those of their affiliated organizations, or those of the publisher, the editors and the reviewers. Any product that may be evaluated in this article, or claim that may be made by its manufacturer, is not guaranteed or endorsed by the publisher.
